# 

**Published:** 1924-03

**Authors:** 


### ORGANISING A HEALTH WEEK.

### A PRIZE OF £100.

"HEALTH Weeks" are a comparatively new departure. Here and there a great town has organised such a week, and there has recently been a very successful one in the Borough of St. Pancras, originated by the Acting Medical Officer of Health, Dr. Stella Churchill. Efforts of this kind are, however, still novel and distinctly occasional. To most people, indeed, the very name is unfamiliar. Yet all manner of "Weeks" and "Days" are constantly being organised for purposes which, however admirable, are mostly of small moment compared with the primary social subject of the health of the people. The State devotes a more minute attention than ever before to the physical well-being of the nation, but we are a sturdy and independent race, and we love to do things for ourselves and not to be beholden to Governments when by individual initiative we can help ourselves. The laws of health must needs be emphasised by the laws of the realm, but there is much that can be done outside Governments to make the sanitary code laid down by Nature better known and more carefully followed. We are only beginning to recognise the imperious character of this code, the observance of which would, in comparatively a few years, add enormously to the health, happiness and well-being of the people.

There is no better, no readier, no more popular means of impressing upon the public mind the duty, social and religious, of doing everything that in us lies—and every intelligent person can do much—not merely to reduce disease, but to prevent it. The medicine of the future will be mainly preventive medicine, and it is to prevention that "Health Weeks" primarily look. To teach the people how to preserve health, how to safeguard infant life, how to push death from disease farther and farther away is a noble object. And that, put into a nutshell, is the object of a "Health Week." Who, then, shall deny that there ought in every community in the country to be such a Week, always full of varied and fascinating interest, and always increasing in scope and attraction ?

It is the conviction of THE HOSPITAL AND HEALTH REVIEW that "Health Weeks" are destined to increase rapidly in number and to exercise a wide and powerful influence over the mind of the people. But they require to be carefully thought out, their details to be considered in the light of experience, and the incidents which compose them arranged with an eye to picturesque appeal. The newspaper, the pictorial poster the lecture, the film, the pageant, wireless, even music and singing can, and ought to be, pressed into the service. A Week of this kind should be a joyous Carnival, an event to be looked forward to with animated expectancy, and looked back upon with all the satisfaction of happy realisation. Instruction should be so imparted that a town or a district should long for the opportunity of acquiring knowledge of the laws of health and how to observe them. A dull or monotonous "Health Week" is certain to be a failure.

In the hope and in the confident expectation of popularising and greatly increasing the number of "Health Weeks" THE HOSPITAL AND HEALTH REVIEW is offering a prize of One Hundred Pounds for the best and most practical answer to the following three simple questions :—

1. How would you start a "Health Week" in your town or district ?
2. What would you make the principal features (naming not more than three) and why ?
3. How would you insure the success of your "Health Week" ?

We do not prescribe any length for these answers. They may be short or long, as the competitor pleases, but it is obvious that the most effective answers are likely to be crisp and compendious. A competitor may send in as many replies as he pleases, but each one must be accompanied by the coupon which will be found at the foot of page 96 of this issue. The competition will remain open until March 25, and we wish it to be clearly understood that it is open to all the world, men and women, doctors and laymen, health visitors and nurses. Everyone who has ideas on the subject is invited to give us the benefit of them. Each answer must bear the name and address of the competitor legibly inscribed. The best answer will be published, together with the name, address and portrait of the winner in our May number, which will be on sale on April 25. We reserve the right to publish any other answer which may be deemed to be sufficiently excellent. The decision of the Editor of THE HOSPITAL AND HEALTH REVIEW is to be accepted as final and conclusive.

**THE HOSPITAL AND HEALTH REVIEW is obtainable of all Newsagents and Booksellers, Price 6d. monthly, or 7½d. post free, from The Publisher, The Hospital Building, 28-29 Southampton Street, Strand, London, W.C. 2.**

### ANSWERS TO CORRESPONDENTS.

S.S.—We know of no book on Cottage hospitals published since the war.

T. L.—You had better consult the Surgical Manufacturing Co., of 83-85, Mortimer Street, W. 1.

M. D. (Canada).—Messrs. Johnson and Sons, 23, Cross Street, Finsbury, E.C. 2; Messrs. Forster and Gregory, Ltd., Streatham Common, S.W. 16.

CONVALESCENT.—Yes, the Sisters have a Convalescent Hospital—St. Andrew's—at Clewer. There is also a Convalescent Home at Folkestone.

ROSE.—You should write at once to Messrs. James Gray and Son, of 89, George Street, Edinburgh, who are offering a limited number of beds at 55s.

READER.—We think you must mean "Post Mortem: Essays, Historical and Medical," by C. MacLaurin (Cape, 7s. 6d. net). It was reviewed in our issue for July, 1923.

F. G.—Dr. Addison first sat in Parliament in 1910 as Liberal member for the Hoxton division of Shoreditch, a seat which he held throughout his Parliamentary career. As first Minister of Health he held office from 1919 to 1921, and was afterwards for a time Minister without portfolio.

### RECENT APPOINTMENTS.

### Medical Officers, Chaplains, &c.

Dick, Major C., M.C., R.A.M.C. (ret.), M.R.C.S., L.R.C.P.; Medical Superintendent of St. Mary's Hospital, Paddington.

Fawcett, Alan W., M.B., Ch.B. (Edin.); House-Surgeon, Jessop Hospital for Women, Sheffield.

Hall, R. J. B., M.B., Ch.B. (New Zealand); Senior Resident Officer Jessop Hospital for Women, Sheffield.

Hoskin, T. Jenner, M.D., M.R.C.P.; Assistant Physician, Royal Free Hospital.

Howell, B. Whitchurch, F.R.C.S.; Consulting Orthopædic Surgeon to Brookscroft Voluntary Infant Welfare Centre, Walthamstow.

Oddy, H. M., M.A., M.B., M.R.C.P.; Assistant Physician to the Belgrave Hospital for Children.

Rankin, J. H., M.B., D.P.H.; M.O.H., Gelligaer, £780.

Thorn, G. St. Clair, M.B., C.B., C.M.G., A.M.S.; Superintendent Edinburgh Royal Infirmary.

Vickery, Colin E., M.B., Ch.M. (Sydney); House-Surgeon, Jessop Hospital for Women, Sheffield.

### Public Health and Hospital Appointments.

Baker, D. A. G.; Assistant Health Visitor, Southgate, N.

Boydell, Alice Maud, Health Visitor Birkenhead; School Nurse and Health Visitor, Leigh.

Crawford, Margaret B.; Child Welfare Nurse, Helensburgh.

Edgar, J. L., A.R.R.C.; Matron, Maltings Farm Sanatorium, Nayland.

Garley, Dorothy; Health Visitor, Somerset County Council.

Hall, Susan, R.R.C.; Matron, Victoria Hospital, Blackpool.

Matthews, Beatrice, Sister Royal Chest Hospital; Matron, Didworthy Sanatorium, South Brent, Plymouth.

Phipps, Amy; School Nurse and Health Visitor, Middlesex County Hospital.

Preston, E. E.; Superintendent School Nurse, Walthamstow.

Tarbutt, E. M., C.D.B., Assistant Health Visitor and School Nurse, Winchmore Hill.

Turner, Clara E., Health Visitor West Riding of Yorkshire County Council; Health Visitor Herefordshire County Council.

Savage, Hilda M., Matron Nantwich and District Cottage Hospital.

Smith, S. E. Street, Health Visitor Wilts. County Council.

Whiteman, May, Matron Torrington Cottage Hospital; Matron Oakdale Cottage Hospital, Blackwood.

### Visit of Foreign Medical Officers.

Twenty-eight doctors from about twenty countries in Europe and South America and the United States have arrived in London on a visit, which will extend over ten weeks, having as its object the study of public health administration in Great Britain. A similar visit was paid to this country early last year, and British representatives have taken part in visits to other countries for a like purpose. The scheme is arranged by the Health Branch of the League of Nations. They will be distributed in small groups over the country for two months. Liverpool, Manchester, Cardiff, Bradford, Edinburgh, Brighton, and Southampton are the centres in which they will have facilities for studying health administration in populous cities and towns, and in the offices of a number of county medical officers and in several urban and rural districts opportunities will be offered of learning much about British public health administration.

"THE HOSPITAL AND HEALTH REVIEW"

### Managerial Notices.

*All letters on Business matters must be addressed to the Manager, "The Hospital and Health Review," 28 and 29 Southampton Street, London, W.C. 2.*

RATES FOR SUBSCRIPTION (payable in advance).

| | | | |
|---|---|---|---|
| Three Months (including Postage) | .. .. | 1s. | 9d. |
| Six Months do. | .. .. | 3s. | 6d. |
| Twelve Months do. | .. .. | 7s. | 0d. |

**It is especially requested that remittances be made by Cheque or Postal Order and not Stamps.**
Subscriptions, which may commence at any time, are payable in advance. Cheques and Post-Office Orders should be crossed Westminster Bank, Covent Garden Branch, and made payable to the Manager of THE HOSPITAL AND HEALTH REVIEW.

***Advertisements.*** Scale of charges and particulars of special positions may be obtained on application to the Manager.

*SUBSCRIPTION FORM.*

*Please send me* THE HOSPITAL AND HEALTH REVIEW *for*............ *months, commencing with your issue of* ............, *for which please find enclosed* ........
*Name* ..........................
*Address* ..........................
..........................
..........................
*Date* ..........................
*To*.......................... *Newsagent.*

